# Genetic elimination of field-cage populations of Mediterranean fruit flies

**DOI:** 10.1098/rspb.2014.1372

**Published:** 2014-10-07

**Authors:** Philip T. Leftwich, Martha Koukidou, Polychronis Rempoulakis, Hong-Fei Gong, Antigoni Zacharopoulou, Guoliang Fu, Tracey Chapman, Aris Economopoulos, John Vontas, Luke Alphey

**Affiliations:** 1 Oxitec Limited, 71 Innovation Drive, Milton Park, Oxford OX14 4RQ, UK; 2 School of Biological Sciences, University of East Anglia, Norwich Research Park, Norwich, Norfolk NR4 7TJ, UK; 3 Faculty of Biotechnology and Applied Biology, Department of Biology, University of Crete, Heraklion, Crete, Greece; 4 Department of Biology, Division of Genetics, Cell and Developmental Biology, University of Patras, Patras, Greece; 5 Department of Zoology, University of Oxford, South Parks Road, Oxford OX1 3PS, UK; 6 The Pirbright Institute, Ash Road, Woking GU24 0NF, UK

**Keywords:** medfly, release of insects carrying a dominant lethal, sterile insect technique

## Abstract

The Mediterranean fruit fly (medfly, *Ceratitis capitata* Wiedemann) is a pest of over 300 fruits, vegetables and nuts. The sterile insect technique (SIT) is a control measure used to reduce the reproductive potential of populations through the mass release of sterilized male insects that mate with wild females. However, SIT flies can display poor field performance, due to the effects of mass-rearing and of the irradiation process used for sterilization. The development of female-lethal RIDL (release of insects carrying a dominant lethal) strains for medfly can overcome many of the problems of SIT associated with irradiation. Here, we present life-history characterizations for two medfly RIDL strains, OX3864A and OX3647Q. Our results show (i) full functionality of RIDL, (ii) equivalency of RIDL and wild-type strains for life-history characteristics, and (iii) a high level of sexual competitiveness against both wild-type and wild-derived males. We also present the first proof-of-principle experiment on the use of RIDL to eliminate medfly populations. Weekly releases of OX3864A males into stable populations of wild-type medfly caused a successive decline in numbers, leading to eradication. The results show that genetic control can provide an effective alternative to SIT for the control of pest insects.

## 1. Introduction

The Mediterranean fruit fly (medfly, *Ceratitis capitata* Wiedemann) is a globally distributed agricultural pest, infesting over 300 types of cultivated and wild fruits, vegetables and nuts—the widest known host range of any pest fruit fly [1]. Control methods include the use of baited traps [2,3], insecticides [4], biological control [5] and the sterile insect technique (SIT) [1,6,7]. Of these, SIT has garnered the most attention as an environmentally friendly and species-specific method, successfully implemented against the medfly as well as several other insects [8]. SIT involves the mass release of sterilized insects to disrupt normal mating patterns and induce a high frequency of sterile matings [9]. A potential benefit of SIT is that it can facilitate the establishment of ‘medfly-free zones’, removing the need for costly quarantine measures that become necessary within medfly-infested areas in order to export produce [10,11].

Overall, SIT must be cost-effective in comparison to other control methods [12–15]. Its success depends upon the ability of released sterile males to find a lek, perform courtship, attract wild females, successfully mate, inseminate females and finally to elicit effective female refractoriness to remating [16–18]. The most common mechanism by which sterility is achieved in SIT released males is through exposure to ionizing radiation. However, this has well-documented negative impacts on longevity and mating performance [16,19–21].

Release of insects carrying a dominant lethal (RIDL) [22–27] provides an alternative that removes the need to subject insects to ionizing radiation. One version of RIDL, on which we focus here, involves the mass release of male insects carrying a female-specific lethal transgene (fsRIDL) [28–31]. Matings with fsRIDL males by wild females produce no viable female offspring, thereby decreasing the reproductive potential of the wild population. If sufficient numbers of females mate with fsRIDL males over time then the population will collapse. Prototype fsRIDL strains of medfly have already been developed [25,27,28,32–34] giving 100% female-specific lethality at the pre-pupal stage. This lethality can be switched off to enable females to develop normally during mass rearing by providing a chemical repressor (tetracycline) in the diet.

OX3864A and OX3647Q are the leading fsRIDL strains for this important agricultural pest. Here, we describe the development and testing of these two new fsRIDL strains, and also large cage population suppression trials using the OX3864A strain. OX3864A provided repressible, fully penetrant female-specific lethality, together with the expression of a dominant fluorescent marker to facilitate identification. In a series of tests of life history, this strain displayed greater fitness than existing strains used for SIT and exhibited a high level of mating competitiveness. Large cage experiments demonstrated proof-of-principle that periodic release of OX3864A males could eradicate wild-type (wt) medfly populations.

## 2. Material and methods

### (a) Medfly stocks

We used the TOLIMAN medfly strain as the wt background for the construction of both transgenic lines and for all subsequent testing of life-history traits prior to the experiments in Crete. TOLIMAN is a wt strain originating from Guatemala; it has been maintained at Oxitec (Oxford, UK) since 2004. *tsl* is the genetic sexing strain T(Y;5)101 called also Vienna-8 (without the pericentric inversion D53), introgressed into the TOLIMAN wt [35]. Genetic sexing in *tsl* is usually achieved by heat stress through exposure of eggs to 34°C for 24 h, though female survival may still be compromised by temperatures above 23°C. A *wp* marker is closely linked to *tsl* and is usually used to detect females [35–37]. OX3864A and OX3647Q are the newly developed RIDL strains tested in this study. Both are engineered to carry DsRed2 fluorescent markers [38] and use the tetracycline-repressible transactivator (tTA), which functions as both the transactivator and lethal effector via a positive feedback loop as described below [27].

### (b) Germ line transformation with OX3864 and OX3647 constructs

Germline transformation of medfly with the OX3864 and OX3647 constructs was carried out using a *piggyBac* transposon vector, by standard micro-injection [39]. Two hundred and thirty pre-blastoderm medfly embryos were microinjected with OX3864, and 2700 with OX3647.

### (c) Insect rearing at Oxitec

Adult fly colonies were kept at 21°C (*tsl* female survival is compromised at higher temperatures), 50% RH and a 13 L : 11 D cycle. Adults were fed on a diet of yeast hydrolysate and sucrose (1 : 4 ratio by mass). Water was supplied through damp cotton wicks. Newly hatched larvae were transferred to larval medium, consisting of standard yeast–wheat germ–glucose–agar diet. For ‘on-tet’ rearing, suppressing transgene expression, this was supplemented with tetracycline hydrochloride to a final concentration of 100 µg ml^−1^. Densities of approximately 1 larva per 0.5 g of diet were used throughout. Pupae were kept between 18 and 25°C (depending on requirements) until eclosion.

### (d) Insect rearing at the University of Crete

Insects were reared as above, except that experiments were conducted at 25°C, and the larval diet comprised 550 ml distilled water, 162 g sugar, 81 g brewer's yeast, 6 g citric acid, 5 g sodium benzoate and 242 g wheat bran. For ‘on-tet’ rearing, this diet was supplemented with tetracycline hydrochloride to a final concentration of 100 µg ml^−1^.

### (e) Tests for longevity

Longevity tests were performed at 21°C and RH 50% in six replicate plastic cages (9 × 9 × 9 cm), each containing 30 males and 30 females of the same genotype (1 insect/8.1 cm^2^ cage surface). Three of the cages were randomly assigned to a stress test of no food and no water. This was done to assess relative measures of nutrient reserves available at eclosion, an important indicator of potential longevity under release conditions. The remaining cages had an ad libitum supply of food and water. Cages were monitored on a daily basis; dead adults were removed and sexed, until all flies were dead, in line with FAO/IAEA/USDA guidelines [40].

### (f) Lifetime female fertility and egg hatching rates

From the non-stressed cages described above, eggs were collected over 24-h periods and counted under a dissecting microscope. The egg samples were then incubated on wet Whatman filter paper (Fisher Scientific) and sealed into a Petri dish with parafilm (200 eggs per Petri dish, 600 eggs per line in total). Seventy-two hours after egg collection, Petri dishes were unsealed and examined under a dissection microscope in order to count the number of empty versus unhatched egg casings.

### (g) Adult eclosion rates

Three hundred pupae from each line were kept singly and monitored for eclosion. Adults were checked for sex and visible deformity before recording. Uneclosed or partially eclosed pupae casings were counted and then discarded.

### (h) Mating competition and remating tests

Competitive mating tests, in which the OX3864A and OX3647Q strains competed against wt males for access to wt females, were carried out according to FAO/IAEA/USDA guidelines [40]. Adult OX3864A, OX3647Q and TOLIMAN wt were obtained from larvae reared ‘off-tet’ at low density (1 larva/0.5 g medium). Field cages (1.25 m tall with a base of 0.5 m^2^) were constructed inside a greenhouse at the Zoology Department, Oxford University (Oxford, UK), with small orange trees (approx. 1 m in height) placed inside. Experiments took place during August (sunrise 06.00) using natural light and a stable temperature and humidity (25°C, 50% RH). Thirty males from either OX3864A or OX3647Q were placed together with 30 wt males at 06.30, and 30 females were introduced 30 min later.

The basic sequence of courtship and copulation is well characterized in the medfly and follows a distinct sequence of male behaviour patterns, consisting of ‘pheromone calling’ and rapid wing vibrations [41]. After courting, the male will leap onto the female and if successful intromission occurs, the pair will generally remain still for the duration of copulation, typically 90–195 min [42]. Mating pairs were removed from cages following intromission, and carefully introduced into horizontally placed 1.5-ml eppendorfs. Copulation initiation time was recorded and copulations were scored as successful only if the pair mated for more than 30 min after transfer to the eppendorf. Short copulations (less than 15 min) were eliminated from the data as they often fail to result in sperm transfer [42]. The mating experiments ended 9 h after initiation (16.00) or whenever all females had copulated, whichever was sooner. The identity of the mating males was determined by scoring for the presence of the DsRed2 fluorescent marker under a fluorescence microscope. Tests were performed with 10 replicates for each line; 167 and 237 couples were assessed for OX3864A and OX3647Q, respectively.

To test the ability of males to induce refractoriness to remating in females, we separated the mated females into two groups of 40 based on their initial mating choice (wt or fsRIDL male) then re-exposed them to equal numbers of wt and fsRIDL males on the following day. This process was repeated with the OX3647Q experiment and run for 3 days, with cages scored for matings for 9 h daily. Mating pairs were removed during mating as described above and the males again genotyped by screening fluorescence. For mating competitiveness tests with wild-derived flies, pupae were recovered from infested oranges gathered from insecticide-free orchards in Heraklion province, Crete. Wild-derived adults were separated by sex immediately after eclosion and left at 25°C, 50% RH for 10–13 days to reach sexual maturity. All flies were allowed to adjust to natural light and temperature conditions of the glasshouse for a minimum of 24 h prior to the start of the experiment. Experiments began 1 h after sunrise and lasted for a minimum of 9 h. Mating tests were performed in greenhouse facilities at the University of Crete. OX3864A mating competition tests were performed in seven replicates with 89 pairs assessed.

### (i) Caged suppression of stable wild-type populations

Stable populations of wt medfly were established in four large field cages with two cages chosen at random to be ‘treatment’ cages into which, in addition to the normal number of pupae added to the cages, approximately 1500 RIDL males per week were released. This protocol was based on that of Valdez *et al*. [30]. The greenhouse-based field cages measured 8 m^3^ and were housed at the University of Crete, Heraklion, using natural light and a stable temperature and humidity (*ca* 25°C, 50% RH). Each cage contained a 1.5 m high lemon tree, three food and water sources and two oviposition pots filled with deionized water (emptied daily), each with two 40 cm^2^ egg laying surfaces.

Wt populations were established over an eight week period by introducing a fixed number of pupae to each cage per week (200 in week 1, 300 in week 2, 180 in week 3 and 230 thereafter in weeks 4–8). Pupal additions for the first four weeks originated from a wt stock colony; thereafter all pupal additions were from eggs caught in the oviposition pots and reared in the laboratory at low density (1 larva/0.5 g medium) before re-introduction to field cages as pupae. Egg numbers were counted daily from the oviposition pots, while adult numbers were calculated weekly (electronic supplementary material, table S1).

At week 7, cages were randomly divided into treatment or control. From week 8 onwards, RIDL treatment cages received weekly additions of 1700 OX3864A pupae reared off-tet (resulting in the addition of approx. 1500 adult males per week). This gave an initial ratio of approximately 7 OX3864A males to 1 wt male in week 8, based on estimates of cage populations (1500 males released into cages with an approximate population of 220 wt males) and a weekly recruitment ratio of roughly 15 : 1 (OX3864A to wt males). Once OX3864A introductions began, the pupal return to a treatment cage was made proportional to its rate of pupae production, with the control cages providing a stable weekly pupal return coefficient for this calculation. For example, in week 16 the mean number of pupae recovered from the control cages was 300. Because returns to the control cages were set at a constant 230 per week, the number of pupae to return to all cages, out of all of those which developed, was set by a coefficient of (230/300 = 0.76). Also in week 16, one of the treatment cages produced a total of 126 pupae. The number of pupae that were returned to this cage (using the coefficient) was therefore 96 (126 × 0.76). This methodology allowed for a dynamic pupal return that was dependent on egg production and pupal survival and reflected the number of eggs laid and the action of RIDL on female larval survival.

### (j) Statistical analysis

Longevity was compared using Cox's model of Proportional Hazards. Comparisons of fecundity and fertility were made using repeated measures ANOVA analysis on (i) total daily egg production per cage, (ii) the estimated mean daily egg production per female and (iii) egg-first instar larva hatch rate. A Greenhouse–Geiger correction was used for egg production per female, to correct for violations of sphericity in the repeated measures design. Comparisons of the eclosion rates and male proportions among lines were made using ANOVA with a Tukey HSD post hoc test. For repeated measures analyses, pairwise comparisons were made following Bonferroni correction. Net reproductive rate per female (*R*_0_) and average generation time (*G*) (spanning the peak of female fertility from one generation to the next) were calculated. From these estimates, an index of fitness (*r*) value for comparison of the potential population growth rate of each line was calculated by adapting the Euler equation [43]. Pairwise comparisons of each relative sterility index (RSI) to wild-derived or wt flies were performed using *t*-tests. Proportions were first arcsine transformed for analysis. Comparisons of remating rates were performed using Fisher's exact tests. The Cox's Proportional Hazards model was run in R [44], all other tests were performed using SPSSv14 (SPSS Inc., Chicago, IL, USA).

## 3. Results

### (a) Strain development

**Figure 1.**
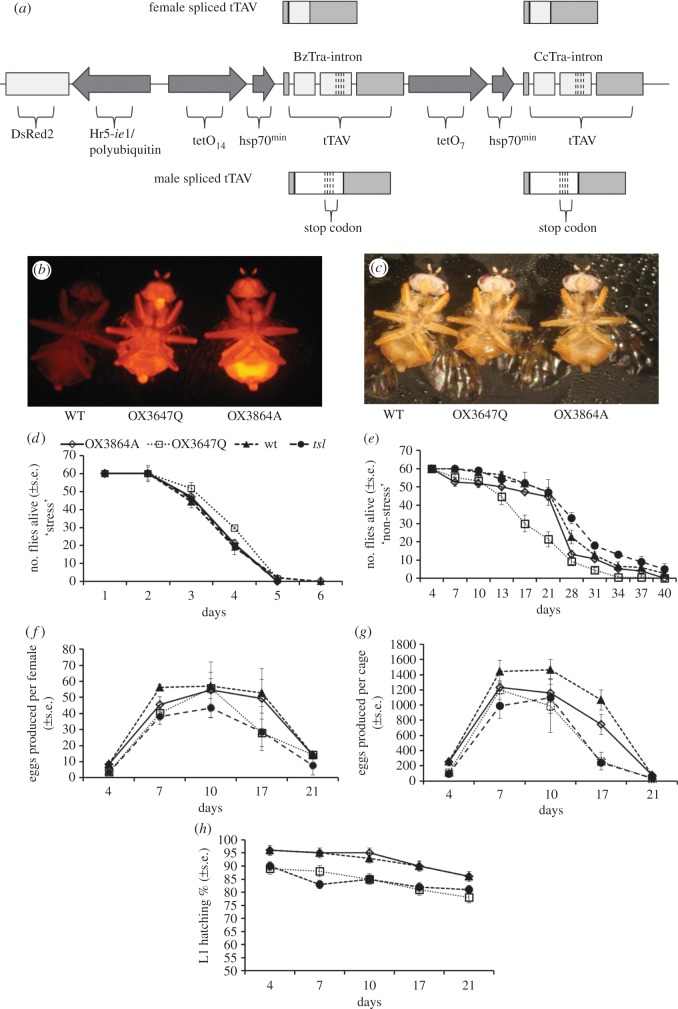
Life-history traits of fsRIDL strain flies, visualization of RIDL transformation marker and RIDL construct details. (*a*) Key elements of transgenic constructs: OX3864 and OX3647 use a tetO–tTA positive feedback system to give tetracycline-repressible lethality [26,31], rendered female-specific by addition of two sex-specific alternative splicing introns from two different tephritid species (to avoid repetitive genetic elements and the risk of recombination): *Bactrocera zonata*, BzTra (GenBank accession: BankIt1696174 BzTra KJ397268), and *Ceratitis capitata*, CcTra [28]. Only females produce splice variants encoding functional tTA protein. OX3864 and OX3647 differ in the promoter used for DsRed2. OX3864 used HR5 IE1, whereas OX3647 used polyubiquitin. (*b*) DsRed2 fluorescence renders the RIDL males (middle, OX3647Q; right, OX3864A) easily and reliably distinguishable from wt (left). (*c*) Same adults as in panel (*b*), under white light. (*d*) Survival under stress test conditions, i.e. without food or water post-eclosion. Adult male and female survival data are combined (*n* = 180). (*e*) Survival under non-stressed conditions of ad libitum food and water (*n* = 180), OX3647Q showed significantly reduced survival relative to wt. (*f*) Female lifetime egg productivity: average production from three cages of 30 females over three weeks. Wt lines produced more eggs than the other three lines; OX3864A produced more eggs than *tsl* and OX3647Q. (*g*) Individual female lifetime fecundity: no difference between the strains in the average number of eggs laid per female. (*h*) Hatching rates of eggs laid by the females in panel (*g*). *tsl* and OX3647Q had reduced egg hatch rates relative to wt, OX3864A did not differ from wt. All values are mean (±s.e.) unless otherwise stated.

Repressible female-specific lethality was achieved by introduction of alternatively spliced, sex-specific introns from the medfly and the peach fruit fly *transformer* genes (*Cctra* and *Bztra*) in the tTA open reading frame [28]. Only females produce functional tTA, and this initiates a lethal tTA positive feedback loop in the absence of tetracycline (figure 1*a*) [28]. This mechanism functions as both the pre-release genetic sexing and post-release population suppression mechanism. The OX3864A and OX3647Q strains developed and tested here contained potentially significant improvements over previous fsRIDL strains. These include double tetO/tTA autoloops with two separate *tra* introns in the open reading frames, and DsRed2 fluorescent markers for ease of identification (figure 1*b*,*c*) [25,28]. *tsl* is the genetic sexing strain T(Y;5)101 called also Vienna-8 (without the pericentric inversion D53), introgressed into the TOLIMAN wt [35].

Forty-nine *G*_0_ adults were obtained for OX3864 (a survival rate of 21%), and 950 for OX3647 (35% survival). Backcrossing to wt flies yielded multiple transgenic lines (electronic supplementary material, table S2). OX3864A and OX3647Q were selected for further development as they displayed complete penetrance, full repressibility, pre-pupal female lethality and excellent fluorescence (table 1). After the initial transformations, the ends of the *piggyBac* transposable elements were removed from the new strains via crossing to strain OX3133, which provides a source of transposase, following the method described by Dafa'alla *et al*. [45] (electronic supplementary material, figure S1). Homozygous lines of each strain were subsequently derived by inbreeding, with confirmation by PCR. Flanking sequences for each insertion site in these two strains are provided in the electronic supplementary material, figure S1, and the chromosomal insertion site of the OX3864 construct in line OX3864A is shown in the electronic supplementary material, figure S2. Insecticide bioassays were conducted (electronic supplementary material, table S3) in order to determine the resistance status of the OX3864A line. We know of no reason for thinking that the transgene might affect insecticide susceptibility, however we considered it important to assess insecticide susceptibility of the strains to minimize the risk that release might introgress insecticide resistance into wild populations.

**Table 1.** Repressibility and penetrance of the female lethality phenotype of OX3864A and OX3647Q on tetracycline and non-tetracycline food. Heterozygous males of each fsRIDL strain were crossed to virgin wt females, with the resulting progeny being reared either ‘on-tet’ or ‘off-tet’. Progeny were scored for sex and fluorescence. There was an equal survival ratio when raised ‘on-tet’, but no transgenic female survivors ‘off-tet’. The ratio of fluorescent to non-fluorescent pupae ‘off-tet’ indicates that constructs induced female lethality at a pre-pupal stage. OX3864A and OX3647Q are independent transgene insertions (electronic supplementary material, figure S1), showing Mendelian inheritance consistent with a single autosomal insertion.

	tetracycline food						non-tetracycline food					
			no. fluorescent adults		no. non-fluorescent adults				no. fluorescent adults		no. non-fluorescent adults	
	no. fluorescent pupae	no. non-fluorescent pupae	♂	♀	♂	♀	no. fluorescent pupae	no. non-fluorescent pupae	♂	♀	♂	♀
OX3864A	351	369	176	160	177	168	60	124	50	0	35	34
OX3647Q	107	88	49	45	36	32	122	302	92	0	125	112

### (b) Longevity

Flies held under the stress conditions had significantly reduced lifespans compared with those provided with food and water (log rank test 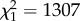, *p* < 0.001). All stressed flies were dead within 6 days (figure 1*d*). In the non-stressed cages supplied with food and water, there were significant effects on survival of strain genotype (i.e. RIDL versus wt and *tsl*; 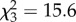, *p* < 0.001; figure 1*e*). Sex had no significant effect on longevity (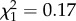, *p* = 0.68) and therefore the survival data for both sexes were combined. Under stress conditions, OX3647Q showed significantly higher survival in both sexes in comparison to the wt (means ± s.e.: wt = 4.1 days ± 0.054; OX3647Q = 4.38 days ± 0.066; Cox's proportional hazards: *z* = −2.1, *p* = 0.035). However, the pattern under full food, non-stressed conditions was reversed (wt = 18.9 days ± 0.52; OX3647Q = 13.7 days ± 0.53; *z* = 5.92, *p* < 0.01). There was no significant difference in the average lifespan of OX3864A and *tsl* flies in comparison to the wt for either the stressed or non-stressed treatments (stressed treatment: OX3864A = 4.13 days ± 0.055; *z* = 0.59, *p* = 0.55, *tsl* = 4.13 days ± 0.064; *z* = 0.53, *p* = 0.33; full food, non-stressed treatment: OX3864A = 17.2 days ± 0.53; *z* = 1.13, *p* = 0.26, *tsl* = 17.0 days ± 0.52; *z* = 0.7, *p* = 0.49).

### (c) Lifetime female fecundity

Per-cage daily egg production from the non-stressed cages declined significantly over time (repeated measures ANOVA: *F*_1.6, 12.9_ = 253.04, *p* < 0.001) and there was also a significant effect of strain genotype (*F*_4.8, 12.9_ = 5.19, *p* = 0.008). Pairwise comparisons with a Bonferroni correction revealed that significantly fewer eggs were produced over the lifetime for both RIDL and *tsl* lines in comparison to the wt (wt mean lifetime egg production = 4315 ± 48.51; OX3864A = 3470 ± 226, *p* < 0.014; OX3647Q = 2593 ± 147, *p* < 0.001; *tsl* = 2465 ± 93.29, *p* < 0.001). OX3647Q and *tsl* strains also produced significantly fewer eggs than OX3864A (OX3647Q versus wt, *p* < 0.001; *tsl* versus wt, *p* = 0.005, figure 1*f*).

By recording daily mortality, it was also possible to estimate age-specific egg production per female. Consistent with the above, this showed that fecundity declined significantly with time (repeated measures ANOVA: *F*_1.9, 15.2_ = 131.85, *p* < 0.001). However, there was no significant effect of genotype on this decline (*F*_5.7, 15.2_ = 2.19, *p* = 0.104, figure 1*g*). Supporting this, a one-way ANOVA on the number of eggs laid at peak fecundity (day 10) also revealed no significant differences in egg laying per female between the lines (*F*_3,8_ = 0.029, *p* = 0.97).

### (d) Egg hatching rates

There was a significant effect of age on egg hatching rates (repeated measures ANOVA *F*_5,40_ = 207.3, *p* < 0.001), as well as a significant effect of strain genotype (*F*_15,40_ = 4.52, *p* < 0.001, figure 1*h*). Pairwise comparisons with a Bonferroni correction showed that OX3647Q and *tsl*, but not OX3864A, had mean percentage egg hatching rates that were significantly lower than the wt (wt = 89.56% ± 0.84; OX3647Q = 79.11% ± 0.84, *p* < 0.001; *tsl* = 78.33% ± 0.84, *p* < 0.001; OX3864A = 87.11% ± 0.84, *p* = 0.247).

### (e) Adult eclosion rates

There was also a significant difference in adult eclosion rates between lines (ANOVA: *F*_3,10_ = 9.89, *p* < 0.001). A Tukey HSD post hoc test revealed that this was mostly attributable to a significantly lower adult eclosion rate in OX3647Q in comparison to wt (wt = 86.1% ± 0.69; OX3647Q = 75.7% ± 2.43, *p* < 0.01; OX3864A = 84.7% ± 0.91, *p* = 0.9; *tsl* = 81.2% ± 1.04, *p* = 0.25). There was a significant effect of strain genotype on adult sex ratio (*F*_3,10_ = 5.06, *p* = 0.036), attributable to a difference in the sex ratio of males to females in OX3647Q but not in the other lines (Tukey HSD post hoc tests: wt = 47% ± 1.8; OX3647Q = 54% ± 1, *p* = 0.035; OX3864A = 55% ± 2.3, *p* = 0.055; *tsl* = 50% ± 1.5, *p* = 0.83).

### (f) Fitness indices

From the individual life-history components, *R*_0_ and *G* were calculated (electronic supplementary material, table S4), and from these estimates an index of fitness (*r*) per female was then derived. The *r* value for the wt was 0.195, which equates to each female contributing on average 0.195 females per day to the next generation. The other lines had lower fitness indices (OX3864A: *r* = 0.187, OX3647Q: *r* = 0.176, *tsl*: *r* = 0.165).

### (g) Mating competitiveness of OX3864A and OX3647 males with wild-type TOLIMAN flies

We used the RSI as a measure of male sexual competitiveness [46]. RSI ranges between 0 and 1. A RSI of 1 would represent 100% of matings by transgenic males, a value of 0 would represent 100% female mating with wt and 0.5 equal numbers with each [40]. The results showed that neither transgenic strain showed a significant reduction in competitiveness relative to wt males (*t*-test: OX3864A: RSI 0.46 ± 0.08, *t*_18_ = −2.09, OX3864A mated males *n* = 77, wt mated males *n* = 90, *p* = 0.05; OX3647Q: RSI 0.47 ± 0.09, *t*_18_ = −1.72, OX3647Q mated males *n* = 112, wt mated males *n* = 125, *p* = 0.1).

We saw no differences in female remating frequency between females initially mated with wt or fsRIDL males (Fisher's exact test: OX3864A: 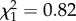, *n* = 40, *p* = 0.775; seven females first mated to OX3864A males remated, eight females first mated to wt males remated; OX3647Q: 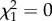, *n* = 40, *p* = 1, 12 females first mated to OX3647Q males remated and 12 females first mated to wt males remated). For those females that did remate when first mated to a RIDL male, no male genotype was preferred (OX3864A: 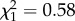, *p* = 0.4 (females that first mated with OX3864A then remated with wt *n* = 3, remated with OX3864A *n* = 4; females that first mated with wt then remated with wt *n* = 5, remated with OX3864A *n* = 3); OX3647Q: 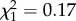, *p* = 0.5 (females that first mated with OX3647Q then remated with wt *n* = 6, remated with OX3647Q *n* = 6, females that first mated with wt then remated with wt *n* = 7, remated with OX3647Q *n* = 5).

### (h) Mating competitiveness of OX3864A males with wild-derived medfly

The mean RSI value of the OX3864A flies when mating with wild-derived medfly from Crete was 0.45 ± 0.13 (*t*-test: *t*_12_ = −0.9, *n* = 89, *p* = 0.38), which gave no evidence for a difference in mating competitiveness between OX3864A and wild-derived males.

### (i) Suppression of stable caged populations of wild-type medfly using OX3864A

**Figure 2.**
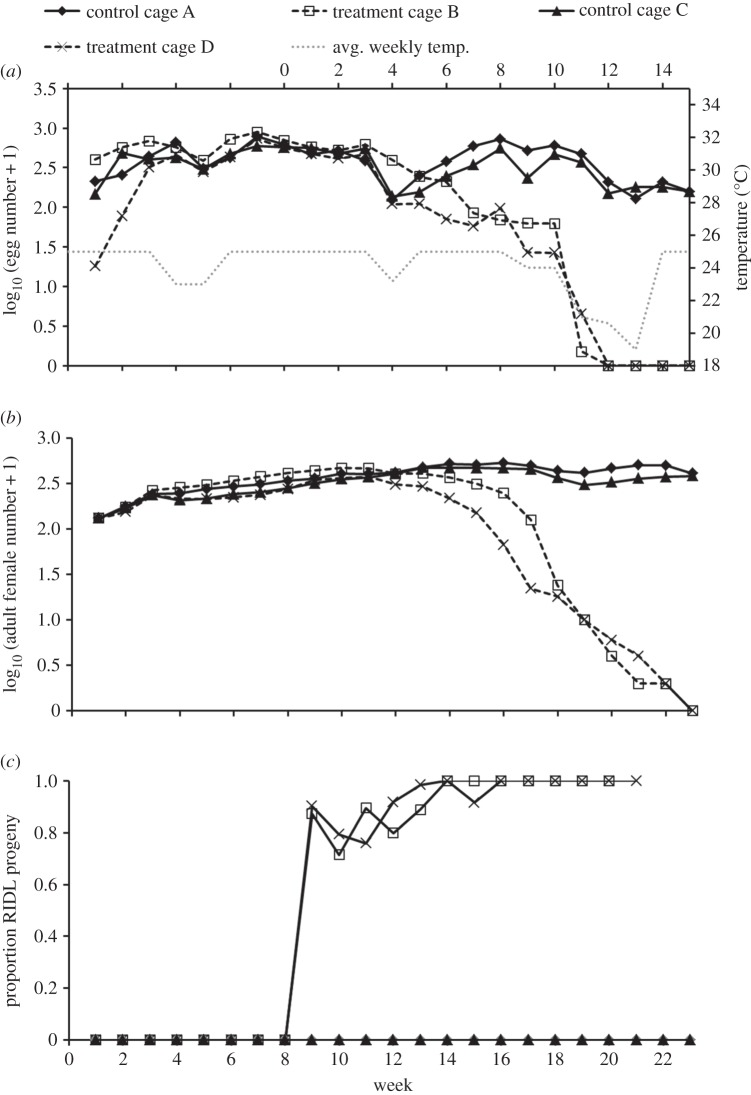
Changing medfly population dynamics through introduction of OX3864A males into large cages of stable wild-type medfly populations. (*a*) Average daily egg production for each week in treatment and control cages. Weeks 0–8 constituted the population stabilization period (230 pupae added each week). Introductions of 1700 OX3864A pupae into each treatment cage commenced from week 8 onwards, in addition pupal return to each treatment cage was made proportional to the control cages. Six weeks after OX3864A male introductions, there was a clear reduction in egg production in the treatment cages compared with the control cages, continuing until eventual extinction of the wild-type population in both treatment cages (as assessed by two weeks of no egg production) by week 22 (14 weeks post initial RIDL release). The dotted line denotes the average weekly daytime temperature (Celsius) taken from daily midday temperature readings. (*b*) Numbers of females in treatment and control cages. (*c*) Proportion of progeny returned to each of the treatment cages from the oviposition traps displaying the DsRed2 fluorescent phenotype. Percentage of returning pupae carrying a copy of the OX3864A transgene reached 100% in both treatment cages by week 17 (11 weeks post-RIDL release).

Dramatic decreases in weekly egg production were observed seven weeks post-RIDL release (PR) in treatment cages, compared with a continued stable rate of egg production in control cages (figure 2*a*). This was due to the proportion of returned progeny carrying the OX3864A transgene increasing in treatment cages, resulting in a rapid decline in the female population (figure 2*b*). Transgene frequency in the treatment cage populations was monitored by screening the returning pupae (chosen from all the pupae produced at random) for the presence of the DsRed2 fluorescent marker. The frequency of the transgene in the returning progeny of the treatment cages was at 100% by week 8 PR (figure 2*c*), with both cages considered extinct by week 14 PR (extinction defined as zero egg production for two consecutive weeks).

## 4. Discussion

In this study, we investigated the OX3864A and OX3647Q homozygous fsRIDL lines of the medfly. Both lines had higher fitness than the classic genetic sexing strain Vienna-8, but equal mating competitiveness with the wt TOLIMAN strain from which they were derived*.* In addition, OX3864A displayed equal mating competitiveness with wild-derived males and proved capable of population suppression in proof-of-principle cage trials. By comparison at each stage with wt and *tsl*, it was possible to investigate any impacts on performance that arose during strain development.

The lack of a strong effect on female survival in the transgenic strains is important as it implies no adverse ‘leaky expression’ of the female-lethal insert at the adult stage. While all the non-wild-type strains displayed reduced egg production over the course of their lifetime when compared with the wt, the magnitude of this effect was greatest for the OX3647Q and *tsl* strains, which were significantly less fecund than OX3864A. However, analysis of age-specific egg production showed no significant differences between any of the strains, indicating that differences in egg production over the lifetime of the cages were due to minor differences in adult female survival rates not previously detected. Pupal to adult eclosion rates did not differ significantly between wt, OX3864A or *tsl*. However, there was a significant reduction in overall eclosion along with a decreased proportion of female eclosion in OX3647Q. This could indicate incomplete suppression of the fsRIDL transgene at this stage.

Mating competitiveness of both transgenic strains was high, neither strain was significantly different from the wt and there was no evidence for negative impacts of transgenesis as measured by this important parameter. In addition, we found no impairment in the ability of transgenic males to induce female refractoriness to remating. By contrast, previous work has shown that female remating is significantly higher following matings to irradiated (as in SIT) as compared with non-irradiated males [21]. Overcoming this deleterious effect by use of an fsRIDL strain represents a significant improvement.

Those negative effects as were observed (e.g. minor decreases in adult longevity and egg hatching rates) could arise from the presence of the transgene, potentially from insertion effects, but could also be due to inbreeding during the construction of the strain [47,48]. Further work could potentially improve the fsRIDL system used here, for example by backcrossing strains to the wt genetic background. This would counter any deleterious effects of inbreeding relative to the wt strain but would not eliminate costs due to the insertion itself. The latter can instead be reduced by the creation and comparison of multiple transgenic lines, as in this study.

Lethality in the OX3864A strain occurs during late larval development. Lethality acting earlier in the life cycle, as has recently been shown in medfly [49] and *Anastrepha suspensa* [50], could be beneficial for control. Such early-acting lethality might reduce factory production costs, as all larvae in the release generation are male and there are no superfluous female larvae competing for food. However, the first and second instar larvae are much smaller relative to the last instar and consume only a small fraction of the total diet required to produce an adult, producing a minimal effect on rearing costs. The stage at which female killing occurs should also have little impact on the potential for fruit damage and efficacy of control. In all sterile-male strategies, including fsRIDL, wild females mating a released ‘sterile’ male still oviposit in fruit, producing ‘sting’ damage which may allow microbial infection. All sterile-male systems aim rather to provide long-term control and damage reduction by reducing the number of females in subsequent generations. Our results show that the fsRIDL system tested has excellent characteristics for this purpose.

The key finding was that both fsRIDL strains tested here showed good competitiveness in life-history tests in comparison to *tsl*. Rearing strains at higher temperatures (not permissive to *tsl*) is predicted to further increase *r* (fitness measured as population growth rates) through a reduction in the generation time (electronic supplementary material, table S4). OX3864A in particular compared well with the TOLIMAN wt and was selected for the next phase of assessment.

There was no evidence for a difference in mating competitiveness between OX3864A and wild-derived medfly under caged conditions. In these experiments, this strain displayed better mating competitiveness (RSI 0.45 ± 0.13) than recorded with traditional genetic sexing strains such as the *tsl* line Vienna-8 (RSI *ca* 0.3 [51]), and far exceeded the minimum proposed RSI for viable SIT strains in tephritids (0.2 [40]). However, larger scale field trials of mating competitiveness are needed to confirm this for release programmes.

Importantly, we have shown that weekly releases of OX3864A males caused rapid, replicated population collapse in stable caged populations. This provides good evidence for the efficacy of fsRIDL. It is conceivable that additional mechanisms besides female lethality could contribute to RIDL population elimination—however, a direct effect of the transgene on egg laying is unlikely to form part of this response because no females survive. Introgression into the target population of maladaptive alleles from the OX3864A strain could occur over time via surviving males. However, this would not be sufficient to contribute significantly to the rapid, massive and sudden population suppression observed in these experiments. This trial used the TOLIMAN wt strain, rather than wild-derived flies (the logistics of establishing new artificially reared populations of wild-derived flies were prohibitive). However, our results suggest that OX3864A would be similarly effective against wild flies. Therefore, fsRIDL appears to present a cheap and effective alternative to the irradiated *tsl* strains currently employed in SIT control programmes (‘on-tet’ rearing may be achieved with an incremental cost of *ca* 1% of the larval diet; electronic supplementary material, table S5). Approval for field studies will be needed in order to make final conclusions. However, we have provided evidence that OX3864A could be used as an effective population control method for the Mediterranean fruit fly.

## Supplementary Material

Supplementary materials

## Supplementary Material

Table S1

## Data accessibility

All datasets supporting this article have been uploaded either as part of the electronic supplementary material or can be found at the Dryad data repository (doi:10.5061/dryad.6sm71).
