# Supplementary material for: Genetic elimination of field-cage populations of Mediterranean fruit flies
Source: Proc Biol Sci. 2014 Oct 7;281(1792):20141372. doi: 10.1098/rspb.2014.1372 (PMC4150327; doi:10.1098/rspb.2014.1372)
Supplement: Supplementary materials [file rspb20141372supp1.docx]

**Genetic elimination of field-cage populations of Mediterranean Fruit Flies.**

**Philip T. Leftwich^1,2^, Martha Koukidou^1^, Polychronis Rempoulakis^1,3^, Hong-Fei Gong^1^, Antigoni Zacharopoulou^4^, Guoliang Fu^1^, Tracey Chapman^2^, Aris Economopoulos^3^, John Vontas^3^ & Luke Alphey^1,5,6^** *

* Corresponding author: [luke.alphey@oxitec.com](mailto:luke.alphey@oxitec.com).

**1** Oxitec Limited, 71 Innovation Drive, Milton Park, Oxford OX14 4RQ, UK

**2** School of Biological Sciences, University of East Anglia, Norwich Research Park, Norwich, NR4 7TJ, Norfolk, UK

**3** Faculty of Biotechnology and Applied Biology, Department of Biology, University of Crete, Heraklion, Crete, Greece

**4** Department of Biology, Division of Genetics, Cell and Developmental Biology, University of Patras, Patras, Greece

**5** Department of Zoology, University of Oxford, South Parks Road, Oxford OX1 3PS, UK

**6** The Pirbright Institute, Ash Road, Woking, GU24 0NF, UK

**Supplemental Figures and Tables**

**Table S1.** See attached Excel spreadsheet

**Table S2.** G2 survival analysis of males and females from different OX lines on- and off-tetracycline. Microinjection survivors (G0) were pooled (either 10 males or 20 females) before being crossed to the TOLIMAN wt. Lines were named according to a number and an alphabetical suffix (e.g. OX3647 Q) to denote the pool from which the G1offspring were collected. Because of the very high number of OX3647 survivors, the alphabet system was re-used and denoted by a number in parentheses before the alphabetical suffix, e.g. OX3647 (2)B. Additional numbers were given to multiple G1 offspring emerging from the same pool (e.g.OX3647 L1, L2) and these were treated initially as potentially separate insertion events. Single transgenic G1 males were each crossed with several virgin wild type female. The G2 progeny were scored for fluorescence (F) or non-fluorescence (NF) and by sex, on tetracycline- (T, 100 µg/ml) or non tetracycline- (NT) containing media. Sex ratio under these two diet conditions was used to assess functionality of the construct in two crucial parameters: 1) Total suppression of female lethality when fed tetracycline 2) Full female lethality in the absence of tetracycline. Lines were selected for further testing based on their ability to meet these parameters and on the strength of fluorescence.

|  | T food | | | | | | NT food | | | | | |
| --- | --- | --- | --- | --- | --- | --- | --- | --- | --- | --- | --- | --- |
|  | Pupae | | F adults | | NF adults | | Pupae | | F adults | | NF adults | |
| Line | F | NF | ♂ | ♀ | ♂ | ♀ | F | NF | ♂ | ♀ | ♂ | ♀ |
| 3647L1 | 61 | 46 | 5 | 7 | 10 | 2 | 106 | 81 | 0 | 107 | 81 | 0 |
| 3647L2 | 89 | 106 | 41 | 41 | 35 | 44 | 63 | 60 | 33 | 37 | 28 | 37 |
| 3647L3 | 307 | 223 | 55 | 232 | 178 | 21 | 87 | 93 | 8 | 82 | 92 | 1 |
| 3647G | 17 | 26 | 11 | 6 | 10 | 15 | 72 | 58 | 16 | 16 | 29 | 27 |
| 3647M1 | 79 | 32 | 23 | 39 | 24 | 0 | 97 | 60 | 6 | 70 | 47 | 0 |
| 3647M2 | 76 | 2 | 27 | 37 | 1 | 1 | 76 | 17 | 39 | 34 | 4 | 12 |
| 3647M3 | 47 | 47 | 0 | 42 | 43 | 1 | 46 | 27 | 0 | 46 | 26 | 1 |
| **3647Q** | **107** | **88** | **49** | **45** | **36** | **32** | **122** | **302** | **92** | **0** | **125** | **112** |
| 3647P | 199 | 199 | 92 | 88 | 84 | 72 | 61 | 96 | 21 | 0 | 24 | 25 |
| 3647(2)B | 138 | 188 | 61 | 46 | 90 | 71 | 207 | 380 | 171 | 0 | 185 | 159 |
| 3647(2)C1 | 139 | 146 | 53 | 66 | 66 | 58 | 105 | 132 | 63 | 0 | 65 | 65 |
| 3647(2)C2 | 196 | 299 | 95 | 83 | 121 | 127 | 152 | 217 | 132 | 0 | 152 | 142 |
| 3647(2)J | 240 | 196 | 133 | 88 | 83 | 84 | 177 | 231 | 108 | 0 | 92 | 102 |
| 3647(2)W | 194 | 168 | 80 | 82 | 66 | 66 | 141 | 185 | 79 | 0 | 84 | 84 |
| 3647(3)C_1_ | 305 | 175 | 125 | 2 | 42 | 50 | 11 | 29 | 8 | 0 | 8 | 12 |
| 3647(3)C_2_ | 271 | 321 | 152 | 0 | 0 | 153 | 49 | 45 | 48 | 0 | 1 | 44 |
| 3647(3)F_1_ | 105 | 150 | 32 | 37 | 44 | 33 | 66 | 79 | 29 | 18 | 33 | 30 |
| 3647(3)F_2_ | 71 | 70 | 16 | 17 | 23 | 13 | 28 | 34 | 10 | 0 | 12 | 15 |
| 3647(3)G | 181 | 185 | 51 | 55 | 50 | 50 | 132 | 63 | 49 | 41 | 30 | 19 |
| 3647(3)H_1_ | 120 | 109 | 49 | 32 | 54 | 39 | 33 | 33 | 13 | 1 | 17 | 8 |
| 3647(3)H_2_ | 124 | 89 | 40 | 40 | 32 | 29 | 1 | 43 | 44 | 0 | 27 | 33 |
| 3647(3)J_1_ | 12 | 23 | 3 | 0 | 12 | 4 | 21 | 19 | 9 | 0 | 7 | 5 |
| 3647(3)J_2_ | 39 | 52 | 12 | 7 | 14 | 8 | 4 | 20 | 4 | 0 | 11 | 9 |
| 3647(3)K | 2 | 1 | 0 | 0 | 0 | 0 | 1 | 5 | 1 | 0 | 2 | 3 |
| 3647(3)O_1_ | 105 | 47 | 18 | 79 | 43 | 0 | 67 | 54 | 28 | 36 | 34 | 0 |
| 3647(3)P_1_ | 1 | 2 | 0 | 1 | 0 | 1 | 0 | 0 | 0 | 0 | 0 | 0 |
| 3647(3)P_2_ | 44 | 66 | 11 | 17 | 13 | 18 | 38 | 62 | 15 | 1 | 18 | 21 |
| 3647(3)Q_1_ | 68 | 96 | 24 | 18 | 17 | 23 | 31 | 52 | 11 | 0 | 18 | 34 |
| 3647(3)Q_2_ | 27 | 13 | 14 | 8 | 5 | 6 | 0 | 0 | 0 | 0 | 0 | 0 |
| 3647(3)R_1_ | 88 | 116 | 38 | 50 | 61 | 58 | 77 | 158 | 20 | 0 | 65 | 44 |
| 3647(3)R_2_ | 38 | 44 | 24 | 0 | 10 | 42 | 1 | 7 | 0 | 0 | 2 | 2 |
|  |  |  |  |  |  |  |  |  |  |  |  |  |
| **3864A** | **351** | **369** | **176** | **160** | **177** | **168** | **60** | **124** | **50** | **0** | **35** | **34** |
| 3864E | 466 | 514 | 191 | 140 | 171 | 154 | 395 | 696 | 212 | 0 | 262 | 236 |

**Table S3.** Insecticide resistance of the OX3864A RIDL medfly strain in comparison to an insecticide susceptible, laboratory reared, medfly strain from Crete (Lab-Crete). Log-dose probit mortality data from topical application bioassays were generated for adult medfly 2-3 days post-eclosion from the Lab-Crete and OX3864A strains, respectively, to active ingredients from four insecticide classes. The pyrethroid, α-cypermethrin, spinosad (spinosyn), dimethoate (organophosphate) and thiacloprid (neonicotinoid), technical grade (>96% purity) were used. Mortality data were analyzed as described in [1]. The dose-mortality responses provide a slope, lethal concentrations (LC) and 95% confidence limits (CL) of the LC for each mortality line. A population is considered to be significantly (*P* <0.05) different in insecticide resistance than another population when there is no overlap of the 95% confidence limits for median lethal concentration (LC_50_). Resistance factors (RF) of the OX3864A strain relative to the reference (Lab-Crete) strain across all four classes of insecticide tested were also calculated. The laboratory reference strain (Lab-Crete) has been reared without exposure to insectides for 20 years and is designated a highly insecticide susceptible strain. These data show that OX3864A does not differ significantly in RF to the Lab-Crete strain and is therefore highly susceptible to insecticides used for medfly control worldwide.

| Compound | *n* | LC_50_ ng/insect  (95% CL) | | Slope ± SE | *χ*^2^ | df | RF |
| --- | --- | --- | --- | --- | --- | --- | --- |
| **α-cypermethrin (pyrethroid):** | | | | | | | |
| Lab-Crete | 96 | 1.01  (0.70-1.58) | 2.19 ± 0.41 | | 3.6 | 12 | - |
| OX3864A | 114 | 0.65  (0.44-0.93) | 2.79 ± 0.54 | | 4.5 | 10 | 0.64 |
| **Spinosad (spinosyn):** | | | | | | | |
| Lab-Crete | 66 | 0.78  (0.48-1.24) | 2.88 ± 0.75 | | 1.6 | 7 | - |
| OX3864A | 93 | 0.92  (0.65-1.34) | 3.65 ± 0.87 | | 2.6 | 8 | 1.18 |
| **Dimethoate (organophosphate):** | | | | | | | |
| Lab-Crete | 60 | 5.02  (3.02-7.60) | 3.4 ± 0.98 | | 2.8 | 6 | - |
| OX3864A | 60 | 5.90  (3.61-9.01) | 3.4 ± 0.94 | | 0.64 | 6 | 1.17 |
| **Thiacloprid (neonicotinoid):** | | | | | | | |
| Lab-Crete | 61 | 35.02  (21.4-56.0) | 2.8 ± 0.73 | | 2.9 | 6 | - |
| OX3864A | 60 | 30.04  (17.8-47.7) | 3.2 ± 0.93 | | 3.3 | 5 | 0.86 |

*n* = Number of medfly tested; LC_50_ = median lethal concentration of insecticide for the population ± 95% confidence limits (CL)^;^ Slope = slope of the dose-mortality response (± standard error, SE); *χ*^2^ = Chi-square test of linearity of dose-mortality responses as described in Roditakis et al. (2005) [1]; df = degrees of freedom for *χ*^2^ tests. All *χ*^2^ tests were not significant (*P* > 0.05) indicating no differences across the two medfly strains in insecticide resistance for any insecticide tested. RF = resistance factor of OX3864A strain relative to Lab-Crete strain (LC_50_ of OX3864A/LC_50_ of Lab-Crete strain)

**Table S4.** Indices of Fitness for strains OX3864A, OX3647Q, wild type and *tsl,* calculated from the life history data.

|  | WT | OX3864A | OX347Q | TSL |
| --- | --- | --- | --- | --- |
| Net Reproductive Rate (R_0_) of Females | 267.6 | 183.7 | 113.1 | 133.1 |
| Generation time in days (G) | 32 | 32.1 | 35.6 | 36 |
| Index of fitness (*r*) | 0.195 | 0.187 | 0.176 | 0.165 |

**Table S5** Costs of tetracycline use when included into standard mass rearing cost analysis. Estimates based on the IAEA business plan [2] which models a large mass rearing facility producing fifty billion flies per year (*tsl* strain) and estimates US$ 5 million per year in diet costs and US$ 375 million per year in additional production costs (excluding cost of capital). Chlortetracycline may be purchased for ca. US$100/kg (EBiochem) and is used at a concentration of 100µg/ml. Use of tetracycline may incur some additional costs e.g. in respect of disposal of spent diet, depending on local regulations and current practice.

|  | Cost of tet used per million flies produced (US$) | Cost of diet per million flies produced (US$) | Production cost of medfly rearing per million flies |
| --- | --- | --- | --- |
| Estimated costs | **1** | **100** | **375** |
| Net cost | **1** | **101** | **476** |
| Percentage cost | **100%** | **0.99%** | **0.21%** |

**Figure S1.** Transgenic insertion sites for RIDL constructs in medfly lines OX3864A and OX3647Q.

Shown are the genomic sequences adjacent to the transgene insertions and the complete removal of *piggyBac* sequences, via methods described in [3] in; **A**) OX3864A-Cca and **B**) OX3647Q-Cca. Flanking sequences are capitalised and were obtained following PCR from the homozygous, *piggyBac* free strains, primer sequences are underlined. Sequences of the constructs (truncated) are shown in lower-case text. PCR was carried out with AttpF1 and adaptor PRIMER, followed by a nested PCR with AttpF2 and MID. The other end of the flanking sequence was obtained following PCR using Flanklox1 and PRIMER followed by a nested PCR with Flanklox2 and MID. PCR fragments were cloned into pJET 1.2 (Fermentas, Thermo Scientific) and sequenced by GATC Biotech Ltd. Sequences were analysed using VectorNTI (Invitrogen).

AttpF2 (CCCAACTGAGAGAACTCAAAGGTTAC),

AttpF1 (GGTCACAACCCCTTGTGTCATGTC),

Flanklox2 (CCTGCAGGGGAGCTCCAGCTTTTG)

Flanklox1 (GGATAACTTCGTATAATGTATGCTATACGAAG)

PRIMER (GTGTAGCGTGAAGACGACAGAA)

MID (GACGACAGAAAGGGCGTGGTG)

A)

CTACATGCGACACATCAGCTACGTAATCTTTTGTATTATTCATTGGTGAGCACCAATATTAAGTGCGCTTATTAGCCGCTATTTTTCTTAATAGCGTGGTTAGTAAGTTGTTTTGTAAGCCTGTATATTTTCAGGCATTAATTGACGGTAAAATGTATAAGCAGGATTAAGTGACAGGCTCAACTTACATATATATTCGTAATTGTTTTTGTGGCCTGTACTACCAGCTTAGGTGAAGCTGGCTGACCCAGTGGGTCTCACATATATGAAATACATCCATAGTGTTACCAGGCACACATACAGCGGGCATTCACCTGTTAAGTGCTATACTCTTTCCATACACTCATTGCATTTTCTACATTTGTAGTCCCCAGTCGACCCTATATAAAACGCGTGAGAGGCCAAAAGGCAATGCCCTGTTAACAGCCCCATCAGCACCCTAGAGTTCGGTTTCTCAACAGATAGACAAACTTTGTGAGTTGGACGAGTTTGAAACGAGTTTGCATACGGGTACGTCATTCTCCCTTCGTTTCCTTGTGTATTTTCGTCATTCTTGTCGGGCAAGCAGAACACAGCTGAGAAACGGCAGTGATACCAAAAGAAAACAATATTGACGTAAAAAACGCAGCTGCAGCGAGCTGCAACGAGTGACAGCAATGATATTCCTTACATAAATTCATAGAATTATTAAGATAAAGTCTTTATTTCGATATTAAAGAGTCCGTTATAATCGCGACTCTTTTGAAGTACAAAGACGTTAAATAAAAAATTAATGTTCGAATCATAATGTTATTGATGTCTAAATGCAGTTGTACCTCGCCGAGTTTTGTTGAATATCTAAAAGGTCTGCTGCTACTGCAAGGTGAGAAGCATCCATTCCAGGCAATCTGCTCCATTAACTAAATTTTCCCTTATAATATGAGGTGCTC**ttaa**aatgaatgtaagcactttattaacgaaatctttgggactaggtcgctaaagggaacaaaagctggagctcccctgcaggataacttcgtatagcatacattatacgagttatcctagagcccgggcgaagttcctatactatttgaagaataggaacttcggaataggaacttctagggaagttcctatactttctagagaataggaacttcggaataggaacttcttcgaacgggagtagtgccccaactggggtaacctttga........................tcgcgctcgcgcgactgacggtcgtaagcacccgcgtacgtgtccaccccggtcacaaccccttgtgtcatgtcggcgaccctacgcccccaactgagagaactcaaaggttaccccagttggggcactactcccgaaaaccgcttctgacctgggaaaacgtgaagccccggggcatccgctgagggttgccgccggggcttcggtgtgtccgtcagtacttaatccgcggttgtcctagtcgac**ttaa**ATAAAAATAATGTAAAGACAGCTTGTATGGGAACATTATTTATATTTTCCATTTTTTTACGTTCTCTGTTATCTGCTACGGAGAAACCGATATAAAATGGCGTTCTACTCGAAAATAAGAACATAAATAAAACGGATAAGCCGTTCAACTGCATTCTTCTTCAATTTGTATGTACCCTGAAGAGAAAGATATGCAAAAAAAAAGGTTGATTATGCTGTTCTTACATTTTGGAACTCGTGCAAATAAGTTGCTTTTCGGTGAAATGGCTAAAATATAATTCAGATCAAAAAAATAAGTAATATAATGTGTGAAAACAATACTTAGCGCAAAAAACTAGCCGTCCGTCGTCGGCCGTTGTCGCTAAGAATTTATGATGAAATAAATCATCACAAACCTTAGCAATGGGCAGCTTGCATTTGTTTGCGCATCCATAAATTTGCCGATGCATTTTGGAACATGATTCATCGTTAAAGTTTGCACAGTTGCATTTAGGAAAGTGTGACAACTGTATAAATGGTTACTTTGCAACTGACGTTTTGGAACTCACCCTTTAGTATTGTATGTTTTACACTATGATTCAATAATTAAAGGTTGGATAATGGGAAGTAGAGGATACAGGCTCCGCTTGAATGGTAAGTTAACCATTGACCAAATATTCACCATGCGGCAAATTTTGGTAAAGACACATGAAAAGGTCGCATTCGACGCCACGAATAGGAGAAAACTTTACGCCGCTATGAATTTGGTATCCCAGCAAACTTATACGACTATGTAACTGACGTTAAGCAACACGAAAGCTCCGTCATGATTGGGGAGATCTCTCGAGCCGGTTCGATACAGACGAGGTATC

B)

tgctgagctgcaggttatgcggtaaagtatttaatttcagctcaggcttgagccatcactacaaagtggcacataacttaaaaaaatcactttcgaaagtgaaatcagaagagtccgttctaatgcctgaggagcttaaaacagaaataatcgatatgaaaacggaaaaagtagagaatgacgaagaattttgtaatataattaaaagtaatgaggaaattagccaaaaggaagaggaggccaagcgagtaattgtggaactgatacaaaatgcaacatatacatcattctttccggaaaactttaatagctctatagattctggtccgccaatagcacgcgaagaaacaatatcagcagtaaacagcattgttagtgaggaaacctcctgaattttaatctatattctatttaagtgtgttatgtactcggtattaatgtaataataattgtagaattataattaacaaaatgtgaaatattatttatataatatgatatttatatagatactgaatgcatctatgtatatatgtacatacat**ttaa**gtcgactaggacaaccgcggattaagtactgacggacacaccgaagccccggcggcaaccctcagcggatgccccggggcttcacgttttcccaggtcagaagcggttttcgggagtagtgccccaactggggtaacctttgagttctctcagttgggggcgtagggtcgccgacatgacacaaggggttgtgaccggggtggacacgtac........................gcactactcccgttcgaagaagttcctattccgaagttcctattctctagaaagtataggaacttccctagaagttcctattccgaagttcctattcttcaaatagtataggaacttcgcccgggctctaggataacttcgtataatgtatgctatacgaagttatcctgcaggggagctccagcttttgttccctttagcgacctagtcccaaagatttcgttaataaagtgcttacattca**ttaa**tcatttctgttattagaaaaagaattatgctgattcgtaattttttatttgcctttatagcaaatttcttgtgaaaaaatcggttgaagttttaattatgaaaaagtaccaagttctttaaaaatttgttaatatgtattaaatctataatccaaattttttcatttataatttaggatagttaattttagaatattaaaaataatttacattgttagaaaaattctgtctgccacattcatgttatttattggcaactctaaaaatttattgtcaaaattgtcaatctaccatctcgaattcgttggcatcggcaaataacacgctgcaactaaatatttattcagttttatttaatccgcaaaaatgcatcccgatcttactgagcgcatattgcaacatttagaaggagttgacaaggtgaacactattgatttagccacactatttggtgttggtcaccaaaaaattgtgggagcattaaaaagtattgaagcccatg

**Figure S1.**

**Figure S2.** Chromosomal insertion site of OX3864 construct in Medfly strain OX3864A. Arrows indicate the hybridisation signal at the 12A position of 2R chromosome arm. Polytene chromosome and probe preparations, also *in situ* hybridizations were as described in Zacharopoulou et al. (1992) [4].


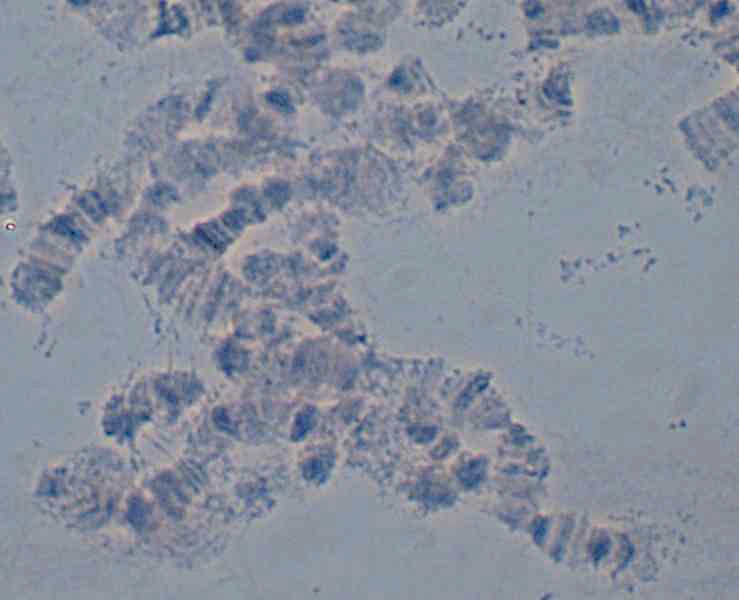

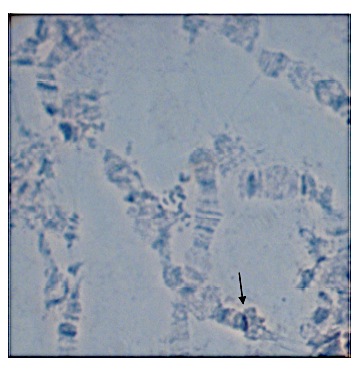


**Figure S2.**

**References**

1. Roditakis E, Roditakis NE, Tsagkarakou A. 2005 Insecticide resistance in Bemisia tabaci (Homoptera: Aleyrodidae) populations from Crete. *Pest Manag Sci* **61**, 577-582.

2. FAO/IAEA 2008. Model Business Plan for a Sterile Insect Production Facility (Vienna, Austria, IAEA) pp. 396

3. Dafa'alla T.H., Condon G.C., Condon K.C., Phillips C.E., Morrison N.I., Jin L., Epton M.J., Fu G.L., Alphey L. 2006 Transposon-free insertions for insect genetic engineering. Nat Biotechnol 24(7), 820-821. (doi:10.1038/nbt1221)

4. Zacharopoulou A, Frisardi M, Savakis C, Robinson AS, Tolias P, Konsolaki M, Komitopoulou K, Kafatos FC. 1992 The genome of the Mediterranean fruitflyceratitis capitata: Localization of molecular markers by in situ hybridization to salivary gland polytene chromosomes. *Chromosoma* **101**, 448-455.
